# Impact of Ozone, Sex, and Gonadal Hormones on Bronchoalveolar Lavage Characteristics and Survival in SP-A KO Mice Infected with *Klebsiella pneumoniae*

**DOI:** 10.3390/microorganisms8091354

**Published:** 2020-09-04

**Authors:** Chintan K. Gandhi, Anatoly N. Mikerov, Faryal Durrani, Todd M. Umstead, Sanmei Hu, Guirong Wang, David S. Phelps, Joanna Floros

**Affiliations:** 1 The Center for Host Defense, Inflammation, and Lung Disease (CHILD) Research, Department of Pediatrics, The Pennsylvania State University College of Medicine, Hershey, PA 17033, USA; cgandhi@pennstatehealth.psu.edu (C.K.G.); am.07@inbox.ru (A.N.M.); faryal_durrani@yahoo.com (F.D.); tumstead2@pennstatehealth.psu.edu (T.M.U.); SAH212@pitt.edu (S.H.); WangG@upstate.edu (G.W.); dphelps@pennstatehealth.psu.edu (D.S.P.); 2 Saratov Hygiene Medical Research Center, The Federal Budget Scientific Institution “Federal Scientific Center for Medical and Preventive Health Risk Management Technologies”, 410022 Saratov, Russia; 3 Department of Microbiology, Virology, and Immunology, The Saratov State Medical University Named after V.I. Razumovsky, 410012 Saratov, Russia; 4 Department of Obstetrics and Gynecology, The Pennsylvania State University College of Medicine, Hershey, PA 17033, USA

**Keywords:** sex differences, oxidative stress, surfactant protein-A, pneumonia, innate immunity

## Abstract

Surfactant protein A (SP-A) plays an important role in innate immunity. The sex-dependent survival of infected SP-A knockout (KO) mice has been observed. Our goal was to study the impact of ozone (O_3_) and sex, as well as gonadal hormones, on the bronchoalveolar lavage (BAL) readouts and survival, respectively, of *Klebsiella pneumoniae-*infected SP-A KO mice. Male and female SP-A KO mice were exposed to O_3_ or filtered air and infected with *K. pneumoniae*. We studied markers of inflammation and tissue damage at 4, 24, and 48 h, as well as the survival over 14 days, of gonadectomized (Gx) mice implanted with control pellets (CoP) or hormone (5α-dihydrotestosterone (DHT) in female gonadectomized mice (GxF) or 17β-estradiol (E_2_) in male gonadectomized mice (GxM)). We observed: (1) an increase in neutrophil and macrophage inflammatory protein-2 levels as time progressed post-infection, and O_3_ exposure appeared to increase this response; (2) an increase in lactate dehydrogenase, total protein, oxidized protein, and phospholipids in response to O_3_ with no consistent sex differences in studied parameters; and (3) a reduction in survival of the GxM and CoP mice, the GxM and E_2_ mice, and the GxF and DHT mice but not for the GxF and CoP mice after O_3_. Without SP-A, (a) sex was found to have a minimal impact on BAL cellular composition and tissue damage markers, and (b) the impact of gonadal hormones on survival was found to involve different mechanisms than in the presence of SP-A.

## 1. Introduction

Pneumonia is the most common infectious cause of childhood mortality globally [1] and a leading cause of death worldwide [2]. *Klebsiella Pneumoniae (K. pneumoniae)*, a Gram-negative enterobacterium, has been recognized as a possible cause of pneumonia, particularly nosocomial infection in individuals with impaired pulmonary defenses [3]. Due to increasing morbidity and mortality of pulmonary infections caused by *K. pneumoniae*, it is important to study the factors that can influence the incidence, susceptibility, and severity of *K. pneumoniae* infection. A number of laboratories including our own have developed mouse models of pneumonia caused by *K. pneumoniae* to understand the mechanisms responsible for host defense against this pathogen and the implications of infection [4,5,6,7]. Furthermore, studies have shown severe *K. pneumoniae* infection in animal models lacking surfactant protein A (SP-A) [4], lysozyme [8], and β 2-microglobulin [9], thus indicating a multifactorial role of host defense against this pathogen.

Pulmonary surfactant is a lipoprotein complex essential for life and normal lung function [10]. The hydrophilic SP-A serves as a first line of host defense for inhaled bacteria and has been shown to play a critical role in innate immune function in the lung [11,12]. Among various immune-related functions, SP-A has been shown to enhance the clearance of pathogens by acting as an opsonin [11,13], participating in the development of dendritic cells [14], regulating the production of cell surface antigens [15], and controlling reactive oxygen species (ROS) [16,17,18]. Deficits in these functions in SP-A knockout (KO) mice makes them more susceptible to pneumonia resulting from various infectious agents such as *Pseudomonas aeruginosa* [19], group B *Streptococcus* [20], *Haemophilus influenzae* [20], and *K. pneumoniae* [4] than wild type mice. Differences in lung function and the severity of lung diseases due to sex have also been observed [5,21,22,23,24,25]. Multiple observational studies in humans have shown an increased incidence and severity of pneumonia in males compared to females [26,27]. Furthermore, an animal model of LPS-induced sepsis showed increased total leukocyte, polymorphonuclear cell, and TNF-α levels in bronchoalveolar lavage (BAL) fluid, as well as greater airway hyper-responsiveness in males compared to females [28]. In contrast, the prior exposure of infected animals to ozone has been shown to increase the susceptibility of females to respiratory diseases compared to males [4,5,29,30,31]. Moreover, in line with human studies, previous studies have demonstrated the decreased survival of both SP-A KO and wild type male mice compared to females after *K. pneumoniae* infection, but the survival pattern reversed following the prior exposure of ozone [4,5]. SP-A KO mice have shown a phenotype deficient in the formation of tubular myelin (TM) in the alveolar surfactant [32,33], TM has been considered as a precursor/reservoir of the surface-active film [34]. However, the underlying mechanism(s) by which SP-A, gonadal hormones, and sex exert these effects are not well-understood.

Ozone (O_3_) can affect SP-A-related functions due to its strong oxidizing ability. In vitro and in vivo studies have shown that ozone-induced SP-A oxidation inhibits its effect on phosphatidylcholine secretion from alveolar type II cells [35,36], reduces its ability to interact with alveolar macrophages (AMs) [35], and has a negative impact on its regulation of cytokine production [37,38]. Moreover, ozone has been shown to decrease the ability of SP-A to stimulate the phagocytosis of both Gram-positive and Gram-negative bacteria, as well as superoxide anion production by AMs [39,40]. After the ozone exposure of SP-A, its aggregation pattern, absorption spectra, gel electrophoretic pattern [41], and SP-A-dependent extracellular surfactant morphology are also changed [35]. Taken together, these data indicate that the oxidation-induced impairment of SP-A activity may be one of the mechanisms that contributes to the increased susceptibility of pneumonia when ozone levels are elevated. Of note, the lower concentration of ozone delivered with oxygen as a vehicle has been used as a therapy [42]. The rationale for this potential therapy is based on the observation that ozone acts as a modulator of NF-κB/Nrf2 pathways [42]. Interestingly, similar pathways and molecules have been affected by SP-A in response to infection and ozone exposure [43], suggesting the role of SP-A along with ozone as a potential therapy for infection and oxidative stress.

In this paper, we built on previous work carried out in wild type mice and extended this to SP-A KO mice. The goal here was twofold: To investigate changes that may occur in the BAL of SP-A KO mice at a relatively early phase of infection and to study the role of gonadal hormones on the survival of *K. pneumoniae*-infected SP-A KO mice with or without prior O_3_ exposure. For the latter, the survival of gonadectomized (Gx) mice with and without hormonal replacement was investigated.

## 2. Materials and Methods

### 2.1. Animals

Male and female SP-A KO mice on the C57BL/6 (Jackson Laboratory (Bar Harbor, ME)) background were bred in the Animal Care Facility of Penn State University College of Medicine under pathogen-free conditions in accordance with approved Penn State University Institutional Animal Care and Use Committee protocols and policies. SP-A KO mice were used at the age of 8–12 weeks. We used a total of 423 mice for this study.

### 2.2. Preparation of Bacteria

*Klebsiella pneumoniae* bacteria (ATCC 43816) were purchased from the American Tissue Culture Collection (Rockville, MD) and then grown and prepared as described previously [5]. Bacteria were grown for 18 h in tryptic soy broth media at 37 °C until they reached the stationary phase. The suspension of bacteria was diluted until the OD_660_ was equal to 0.4. We used a 200 µL aliquot of this dilution to inoculate 50 mL of fresh media for sub-cultivation for 3 h, resulting in a culture that was in the mid-log phase of growth. We then placed the sub-culture on ice to stop growth. Using cold PBS, the culture was serially diluted to obtain ~9 × 10^3^ CFU/mL, and mice were infected by intratracheally injecting 50 μL of this bacterial suspension (containing ~450 CFU). The CFU per mL values were calculated from the OD_660_ of the bacterial suspension, and an aliquot was also spread on tryptic soy agar plates to confirm CFU estimates.

### 2.3. Exposure of Mice to Ozone and K. pneumoniae Bacterial Infection

Mice were exposed to O_3_ (2 ppm for 3 h) or to filtered air (FA; control) at the same time in separate chambers, as previously described [5]. Each experiment in this work consisted of 10 mice (5 exposed to FA or to O_3_). Mice were infected immediately after exposure, as described previously [5]. Briefly, the animals were anesthetized, the trachea was surgically exposed, and ~450 CFU/mouse were inoculated intratracheally in 50 μL of PBS. If any mice died within the first 12 h post-infection, we considered the death to be related to the surgical procedure rather than resulting from the infection, and those mice were excluded from the study. In cases where mice were moribund with no chance of recovery, the mice were euthanized to prevent unnecessary suffering according to Penn State University Institutional Animal Care and Use Committee recommendations and were included with the natural deaths. After exposure to FA or O_3_ and subsequent infection, mice were subjected to various analyses, as described below.

### 2.4. BAL Analyses

For these experiments, the lungs of the mice were subjected to BAL (3 times with 0.5 mL of 0.9% NaCl) at the 4, 24, and 48 h post-infection time points, as previously described [44]. Three independent experiments were performed for each time point (4, 24, and 48 h). A total of 80 male mice (41 FA-exposed and 39 O_3_-exposed) and 86 female mice (45 FA-exposed and 41 O_3_-exposed) were used. The BAL fluids were centrifuged and the cell pellets resuspended in 0.9% NaCl. Cell-free supernatants were frozen at −80 °C until subsequent analyses were performed. Each mouse BAL sample was subjected to 6 independent cell and biochemical analyses, such as measurements of total protein, total oxidized protein, lactate dehydrogenase (LDH), total phospholipid, and macrophage inflammatory protein-2 (MIP-2) concentrations. The percentage of differential cell counts was measured at 4, 24, and 48 h post-infection. All of these analyses were performed as previously described in detail [30].

### 2.5. Gonadectomy and Hormone Treatment

All animal procedures, including gonadectomy, hormone treatments, O_3_ exposure, and *K. pneumoniae* bacterial infection of SP-A KO mice were done as described previously [24]. Gx females received DHT (dihydrotestosterone) and Gx males received E_2_ (17β-estradiol). Briefly, male and female mice were gonadectomized (GxM and GxF, respectively). One week later, control pellets (CoP) or hormone pellets were subcutaneously implanted (E_2_: 0.006 mg/pellet; DHT: 5 mg/pellet) on the lateral neck between the ear and shoulder. One week after pellet implantation, mice were exposed to either FA or O_3_, and then they were infected with *K. pneumoniae*. The survival of the Gx mice was checked daily up to day 14 of post-infection. Five individual experiments in each experimental group were performed for survival. In total, 98 female mice (49 FA-exposed and 49 O_3_-exposed) and 99 male mice (49 FA-exposed and 50 O_3_-exposed) were used for survival study.

### 2.6. Phagocytosis Assay

The experimental design of in vivo phagocytosis was the same as described above for the survival study with the exception of the bacterial dose (~1.2 × 10^7^ CFU/mouse in 50 μL of PBS). The lungs were lavaged (3× with 0.5 mL of 0.9% NaCl) one hour after infecting the Gx male and female SP-A KO mice to harvest alveolar macrophages. If a mouse died within 1 h of infection, this mouse was excluded from analysis. Alveolar macrophages were prepared as described previously [45] and applied to slides using a cytocentrifuge. The slides were stained using the Hema-3 Stain Kit for analysis by light microscopy. The phagocytic index was calculated as described previously [46]. The actual values calculated for the phagocytic index, rather than percentages or normalized values, were used for this analysis.

### 2.7. Statistical Analysis

All data from BAL analysis were analyzed with a simple *t*-test. Kaplan–Meier survival curves were analyzed at the end of the 14-day period using a log-rank test and the difference in daily survival was analyzed using Fisher’s exact test. Mean survival, standard deviation, and standard error were calculated for five independent experiments for each group (*n* = 5 mice/group). A simple *t*-test and one way ANOVA with post hoc Tukey’s test were used to compare the mean survival of two groups and multiple groups, respectively. The *p* ≤ 0.05 was considered statistically significant.

## 3. Results

We performed two major groups of experiments: (1) we measured a number of parameters to study sequential changes in the BAL of SP-A KO male and female mice at 4, 24, and 48 h after exposure to either O_3_ or FA and *K. pneumoniae* infection, and (2) we measured the effect of gonadal hormones on the survival of *K. pneumoniae*-infected SP-A KO mice after exposure to O_3_ or FA.

### 3.1. BAL Content

#### 3.1.1. Percentage of Polymorphonuclear Leukocytes (%PMNs)

**Figure 1:**
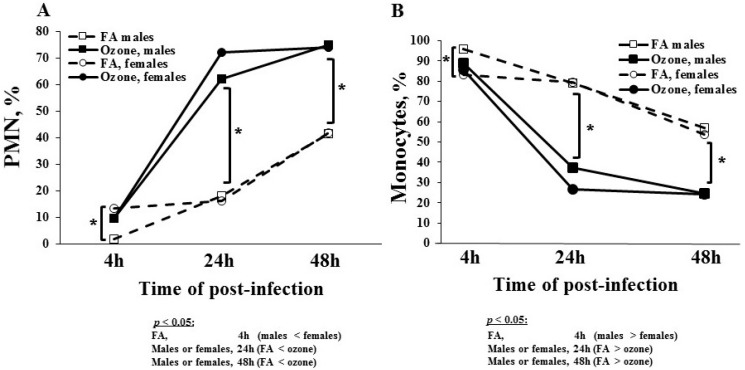
Polymorphonuclear leukocytes and monocytes in the bronchoalveolar lavage (BAL) of filtered air (FA)- and ozone-exposed knockout (KO) male and female mice. Male and female mice were exposed to ozone (or to FA as a control) first, and then they were infected with *Klebsiella pneumoniae* bacteria, as described in the Materials and Methods section. The BAL analysis was done at 4, 24, and 48 h post-infection. Time elapsed post-infection is shown on the x-axis. Males are shown with square boxes, and females are shown with circles. Open shapes and dashed lines represent FA exposure. Solid shapes and lines represent ozone exposure. BAL cells from male and female mice were counted with a hemocytometer. After performing a differential cell count on cytospin preparations, the % polymorphonuclear leukocytes (PMNs) and %monocytes were determined and are graphed on the y-axis in panels (**A**) and (**B**), respectively. Brackets and * indicate groups that differ significantly from one another (*p* < 0.05).

There was a progressive increase in %PMNs (percent polymorphonuclear leukocytes) as the time lapsed after infection in both FA- and O_3_-exposed mice (Figure 1A) in both sexes. In FA-exposed mice, the increase in the %PMN cell count was similar in both males and females at 24 and 48 h post-infection; however, unlike in males, females showed increasing %PMNs to 10% as early as 4 h post-infection. The %PMNs reached ~40% at 48 h post-infection in both sexes.

Similar to FA-exposed mice, there was a progressive increase in %PMNs in O_3_-exposed mice at 4, 24, and 48 h after infection in both sexes. There was a significant effect of ozone on %PMNs at the 24 and 48 h time points in both sexes. At 24 h post-infection, the %PMNs increased from 10% at 4 h to ~65–70%. The %PMNs reached ~75% at 48 h post-infection in both sexes.

#### 3.1.2. Percentage of Monocytes

The change in monocytes was the exact opposite of that seen in PMNs in response to infection and ozone exposure (Figure 1B). We observed a progressive decrease in the monocytes in both FA- and O_3_-exposed mice in both sexes at various time points (Figure 1B). In FA-exposed mice, there was no difference in the %monocytes between sexes at 24 and 48 h post-infection. There was a significant decrease in the percentage of monocytes in females compared to males at 4 h. The percent of monocytes declined to ~55% at 48 h from ~80% to 90% at 4 h after infection.

In O_3_-exposed mice, there was a greater decrease in monocytes at each time point in both sexes. Similar to %PMNs, ozone had greater impact on monocytes at the 24 and 48 h time points. There was no significant difference in the %monocytes between sexes at all time points. The %monocytes was ~85–90% at 4 h and gradually decreased to ~25% at 48 h after infection.

We did not observe significant changes in lymphocytes due to a lower number of lymphocytes in BAL at all time points in both FA- and O_3_-exposed mice in both sexes (data not shown). Collectively, these data showed that although infection had an impact on %PMNs and %monocytes in BAL, prior ozone exposure significantly changed the impact of infection on the different cell types.

#### 3.1.3. Total BAL Protein

**Figure 2:**
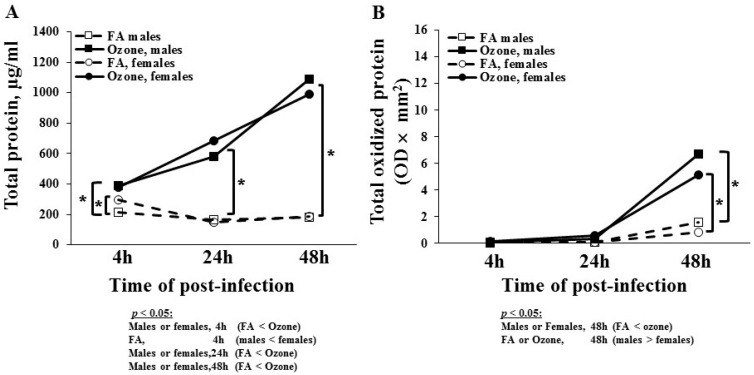
Total and oxidized BAL protein after FA and ozone exposure. Experimental design is presented in the legend for the Figure 1. Panel (**A**) depicts the total protein level in BAL samples, and values (μg/mL) are graphed on the y-axis. The protein content of the BAL fluid was similar at all time points in FA-exposed mice. Meanwhile, the protein content was increased at 24 and 48 h in ozone-exposed male and female mice. Panel (**B**) depicts the total oxidized protein level in BAL. The total oxidized protein level was determined by treating an aliquot of BAL protein with the OxyBlot Oxidized Protein Detection Kit, and the densitometric values (OD × mm^2^) are graphed on the y-axis. Total oxidized protein increased 48 h after infection in ozone-exposed male and female mice. Brackets and * indicate groups that differ significantly from one another (*p* < 0.05).

FA-exposed males had a significantly lower protein content at 4 h than female mice (Figure 2A). There were no sex differences in FA-exposed mice at 24 and 48 h. Though not statistically significant, there was a small decrease in total protein content in FA-exposed mice in both sexes after the 4 h time point. As observed in differential cell counts, O_3_ had a significantly greater impact on total protein content in both sexes at all time points, and with time a progressive increase in protein content was observed in O_3_-exposed mice. Though small differences were observed between males and females at the 24 and 48 h time points, none of these changes were significant. There was no sex difference in O_3_-exposed mice at 4 h. These data showed that infection alone, with the exception at the 4 h time point, had no significant effect on the total BAL protein content, but prior ozone exposure resulted in a significant increase in the total BAL protein content in both sexes.

#### 3.1.4. Total Oxidized BAL Protein

The level of BAL protein that had undergone oxidation in response to infection and ozone is shown in Figure 2B. There was no change in oxidized BAL protein at 4 and 24 h in both sexes in the FA- and O_3_-exposed mice. Though not statistically significant, there was a slight increase in the total oxidized protein level at 48 h in FA-exposed mice. In O_3_-exposed mice, there was a statistically significant increase in oxidized protein at 48 h post-infection. This increase was more pronounced in males than females. These data indicated a significant impact of ozone on protein oxidation in the BAL of SP-A KO mice in both sexes.

#### 3.1.5. Lactate Dehydrogenase (LDH) Levels

**Figure 3:**
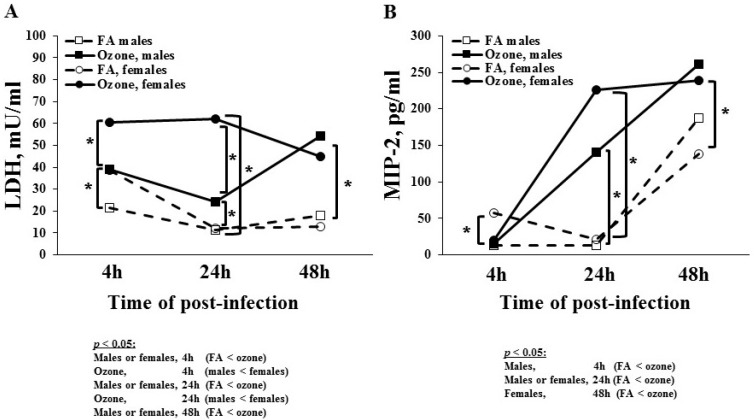
Lactate dehydrogenase (LDH) and macrophage inflammatory protein-2 (MIP-2) levels in BAL. Panel (**A**) depicts the LDH levels in BAL samples from male and female mice. Values (mUnits/mL) are graphed on the y-axis. Panel (**B**) depicts the MIP-2 levels in BAL fluid. Values (pg/mL) are shown on the y-axis. The experimental design is presented in the legend for Figure 1. Brackets and * indicate groups that differ significantly from one another (*p* < 0.05). Statistically significant differences are shown below the Figure.

To study tissue damage, we measured LDH levels in BAL fluid (Figure 3A). In FA-exposed mice, females had higher LDH levels than males at 4 h after infection, but no statistically significant differences were observed at 24 and 48 h in both sexes. In O_3_-exposed mice, the LDH levels were significantly higher compared to FA-exposed mice in both sexes at each time point studied. The O_3_-exposed females had higher LDH levels compared to males at the 4 and 24 h time points. In O_3_-exposed females, LDH levels remained the same at 4 and 24 h and slightly decreased at 48 h. In O_3_-exposed males, LDH levels decreased at 24 h and then increased at 48 h. No significant difference was observed between males and females at the 48 h time point.

#### 3.1.6. MIP-2 Levels

MIP-2 is a neutrophil chemoattractant, and its levels were studied in BAL (Figure 3B). In FA-exposed mice, females had higher MIP-2 levels than males at 4 h; however, no statistically significant sex difference was observed at 24 h. MIP-2 levels increased at 48 h in FA-exposed mice in both sexes. In O_3_-exposed male mice, there was a gradual increase in MIP-2 levels at 24 and 48 h post-infection compared to FA-exposed mice at the same time points. The O_3_-exposed female mice had a robust increase in MIP-2 levels at 24 h—more than that of the male mice, albeit not statistically significant. Similarly, there was no sex difference in the MIP-2 levels at 48 h in O_3_-exposed mice. Thus, infection alone increased MIP-2 levels at 48 h in both sexes, but ozone exposure increased the levels higher and earlier (~24 h), thus indicating a potential role of MIP-2 in O_3_-induced oxidative stress.

#### 3.1.7. Total Phospholipids Level

**Figure 4:**
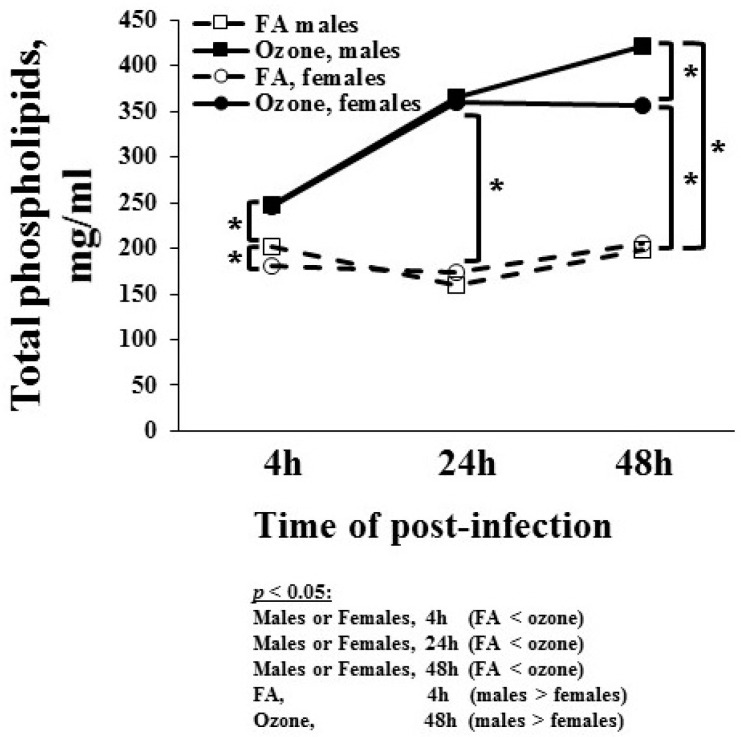
Total phospholipid levels. The phospholipid content of BAL was determined in males and females using the Phospholipids B assay. Values (µg/mL) are graphed on the y-axis. The experimental design is presented in the legend for Figure 1. Brackets and * indicate groups that differ significantly from one another (*p* < 0.05). Statistically significant differences are shown below the figure.

Surfactant phospholipid levels in BAL at different time points in response to infection and ozone were studied and are shown in Figure 4. FA-exposed male mice had higher phospholipids levels than female mice at 4 h. There was no sex difference in total phospholipid levels in FA-exposed mice at 24 and 48 h. In O_3_-exposed mice, the phospholipid levels were significantly higher than in FA-exposed mice in both sexes at each time point. Thus, infection alone had no effect on phospholipid levels. Similar to the MIP-2 levels, O_3_-exposed male mice showed a gradual linear increase in phospholipids levels at 24 and 48 h post-infection, whereas O_3_-exposed female mice showed increased levels at 24 h but no change in phospholipid levels at 48 h. Levels from O_3_-exposed males were significantly greater than those of females at 48 h (Figure 4).

### 3.2. In Vivo Phagocytosis of K. pneumoniae by AMs

**Figure 5:**
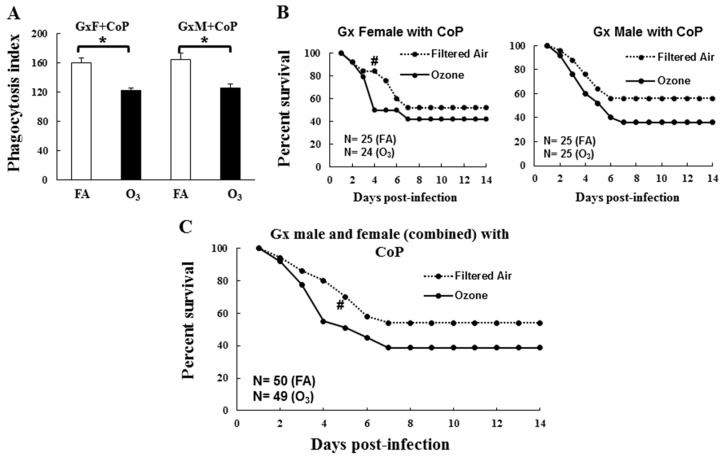
Effect of ozone exposure on survival and in vivo alveolar macrophages phagocytic index of gonadectomized surfactant protein A (SP-A) KO mice after *K. pneumoniae* infection. Experimental design is described in the Materials and Methods section. Panel (**A**) shows the phagocytic index of alveolar macrophages (AMs) from gonadectomized (Gx) male and female mice. Absolute data were used for this analysis and are shown on the y-axis. Bracket and * sign shows significant differences above the corresponding bars (*p* ≤ 0.05). Panel (**B**) shows the survival of Gx male and female mice treated with control pellets (CoP). Panel (**C**) shows the survival of Gx mice (combined male and female) treated with CoP. Animals were monitored for survival up to 14 days after exposure to O_3_ or FA and infection. Significant differences in daily survival were indicated with “#”, *p* ≤ 0.05.

The AMs from O_3_-exposed male and female Gx SP-A KO mice showed significantly lower phagocytic activity compared to their FA-exposed counter parts (females = 122 vs. 160; *p* = 0.002, males = 125 vs. 164; *p* = 0.0005; see Figure 5A). There was no significant sex difference in FA- or O_3_-exposed Gx KO mice.

### 3.3. Impact of Gonadal Hormones on Survival

To distinguish the effects of circulating gonadal hormones on survival from the potential effects of sex-dependent anatomic and physiologic airway differences, Gx females received DHT and males received E_2_. No significant differences in survival were observed between Gx males and females either in response to FA or O_3_ exposure (Figure 5B). The only significant difference was in daily survival on day 4, where O_3_-exposed Gx females showed a lower survival rate compared to FA-exposed females; *p* = 0.02.

**Figure 6:**
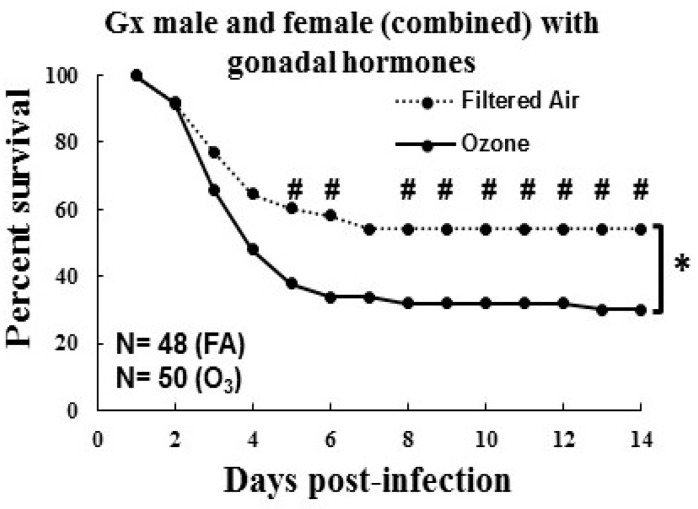
Effect of ozone exposure on the survival of gonadectomized SP-A KO mice with hormone treatment after *K. pneumoniae* infection. Survival of Gx mice (combined male and female) with hormone pellets (dihydrotestosterone (DHT) in GxF and E_2_ (17β-estradiol) in GxM) is shown. Infected animals with prior FA or O_3_ exposure were monitored for 14 days. Bracket and * sign shows significant differences in survival after 14 days, and # sign shows significant differences in daily survival (*p* ≤ 0.05).

Since no sex differences were observed either in the survival or phagocytic indexes, we combined males and females for subsequent analyses. When males and females were analyzed together, O_3_-exposed animals showed a trend of lower survival (*p* = 0.06) compared to FA-exposed Gx mice, although this did not reach significance except for on day 4 (*p* = 0.009; Figure 5C), which was similar to that seen with the Gx females (Figure 5B). However, the treatment of Gx mice with hormones (DHT in GxF and E_2_ in GxM) showed a significantly decreased survival in O_3_-exposed, infected SP-A KO (male and female combined) mice compared to FA-exposed, infected mice (*p* = 0.01, Figure 6). A lower daily survival was observed in O_3_-exposed hormone-treated Gx mice compared to FA-exposed mice over days 5–14, except for day 7, thus indicating a role of gonadal hormones in survival following ozone exposure.

**Figure 7:**
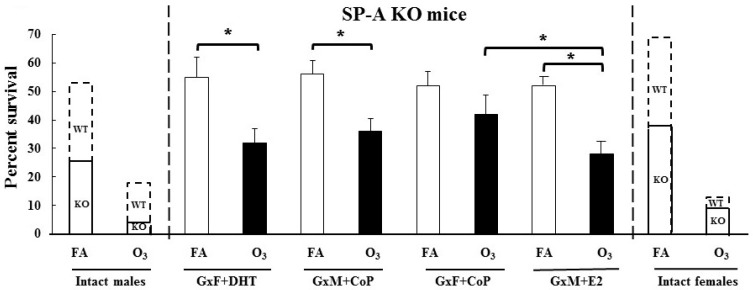
Mean survival of ozone- or FA-exposed gonadectomized and hormone-treated SP-A KO mice after *K. pneumoniae* infection. Gonadectomized SP-A KO female mice were treated with DHT hormone, gonadectomized male mice were treated with E_2_, and the mouse survival after subsequent ozone exposure and *K. pneumoniae* infection was analyzed at 14 days post-infection, as described in the Materials and Methods section. Significant differences (*p* < 0.05) are shown with brackets and * sign on top of the corresponding bars. GxF and DHT mice or GxM and E_2_ mice—gonadectomized females treated with DHT hormone or gonadectomized males treated with E_2_ hormone, respectively. Bar shows mean ± SEM (*n* = 25/group). White and black bars represent FA- and O_3_ exposure, respectively. Data of the current study of Gx SP-A KO mice are shown in between two dotted lines. Survival data of intact (non-gonadectomized) SP-A KO (bottom of the bar with solid lines) and wild-type (WT) (top of the bar with solid dotted lines) mice on extreme right and left from our previous work [4] are presented here for illustrative purposes.

The mean survival of Gx mice was investigated to gain insight into the impact of O_3_ and/or gonadal hormones (Figure 7). The survival of the GxF treated with CoP did not differ between FA- and O_3_-exposed mice (52% vs. 42%, *p* = 0.3). In contrast, significant differences were observed between the FA- and O_3_-exposed GxM and CoP mice (56% vs. 36%, *p* = 0.03) or the GxM and E_2_ mice (54% vs. 28%, *p* = 0.008). The O_3_-exposed GxF mice treated with DHT also showed a significantly decreased survival rate compared to the FA-exposed, DHT-treated GxF mice (55% vs. 32%, *p* = 0.007). There was no difference in the mean survival rate among FA-exposed GxF mice with or without DHT or GxM mice with or without E_2_. In response to O_3_, although there was no significant difference between survival of the GxM and CoP mice and the GxF and DHT mice, a significant difference was observed between the GxF and CoP mice and the GxM and E_2_ mice. Similar results were observed when the survival of the FA- and O_3_-exposed GxM and CoP mice and the GxF and CoP mice were compared to the GxM and E_2_ mice and the GxF and DHT mice, respectively.

Collectively, these data indicated that O_3_ exposure reduced the survival in the SP-A KO GxM and CoP mice and the GxF and DHT mice. However, the GxF mice treated with CoP did not show any difference in the survival between FA- and O_3_-exposed mice, as shown previously with GxF WT mice [24], but the GxM mice treated with E_2_ showed a significant decrease in survival with prior O_3_ exposure. Moreover, sex hormone replacement did not reduce survival rates in Gx male and female-infected mice with or without prior ozone exposure, similar to those observed for intact SP-A KO mice (i.e., non-Gx). This observation, along with the observation that the sex hormone replacement of Gx WT mice resulted in survival rates nearly identical to those observed in intact WT mice [24], indicated that in the absence of SP-A, the effect of gonadal hormones on survival occurs through different mechanisms/pathways than those in the presence of SP-A.

## 4. Discussion

SP-A plays a role in several immune cell functions, the regulation of inflammation, and processes related to lung injury and repair [12]. Previous studies have shown the decreased clearance of various bacteria and viruses, as well was the increased severity of disease in SP-A KO mice [4,20,47,48]. Ozone, one of the major air pollutants, has been shown to oxidize SP-A and affect its function. Moreover, lung immune function has been shown to be affected by sex-specific mechanisms. Our goals in the current study were to: (1) study BAL factors that may contribute to the effect of O_3_ and *K. pneumoniae* and (2) to study the impact of gonadal hormones on the survival of SP-A KO mice.

As anticipated, both infection and ozone exposure led to an increase in markers of lung inflammation and tissue damage. As shown with other mouse models of bacterial lung infection [49], we observed a progressive increase in neutrophils and MIP-2, a potent neutrophil chemoattractant (a homolog of human IL-8 in rodents) secreted by macrophages, in the BAL after infection, with greater increases observed in the O_3_-exposed mice. The percent increase of PMNs observed in SP-A KO was similar to that observed in WT mice (under identical conditions) [30] and in another published pneumonia mouse model [49]. Though the MIP-2 level increased in SP-A KO mice in response to infection and O_3_, the response was much more robust (~700% more) in WT mice, particularly after O_3_ exposure [30]. The robust response in WT mice, as opposed to SP-A KO, indicated that an SP-A-mediated *priming* of macrophages may be a major regulator of MIP-2. Moreover, the phagocytic activity of the alveolar macrophages of SP-A KO has been shown to be reduced compared to WT mice, and O_3_ exposure further reduces this activity in each sex [4,5], indicating that the alveolar macrophages may be functionally hypoactive and lack readiness to respond in the face of a challenge (e.g., infection and oxidative stress) in the absence of SP-A *priming* [50]. Conversely, *K. pneumoniae* infection alone had no or minimal impact on markers of tissue damage such as LDH and total protein levels in BAL, but a prior O_3_ exposure often showed a progressive increase in these markers. Moreover, there was an increase in the total oxidized protein content following O_3_ exposure starting at a later stage of infection (~48 h) compared to WT mice where there was a gradual increase in the total oxidized protein content as early as 4 h. These findings were similar to those of previous studies of ozone-induced lung damage where the effects of O_3_ alone (i.e., in the absence of bacterial infection) on WT and SP-A KO mice were studied [44]. It is of interest that the total protein content in the BAL of SP-A KO mice (present study) was similar to that of WT mice at all time points, but the total oxidized protein content was lower and the rise occurred later (~48 h) in SP-A KO mice (present study) compared to WT mice under similar conditions [30]. This indicates that in the absence of SP-A, ROS production is both reduced and delayed following O_3_ exposure. Moreover, the lack of an immediate rise in oxidized protein following ozone exposure indicated that oxidation may not be a direct result of ozone exposure; instead, it may be a somewhat delayed secondary process resulting from ROS production by activated immune cells. As mentioned above, another possibility is that macrophages do not produce as much ROS in the absence of SP-A-mediated *priming*—hence the lower levels of oxidized protein in the KO mice compared to WT mice. In fact, SP-A KO mice are deficient in superoxide radical generation [51], and this may, in part, explain the reduced clearance of pathogens in the absence of SP-A [51,52]. With the exception of few sex differences, none of the above parameters showed consistent sex differences, as males and females exhibited similar levels and similar changes. Therefore, we concluded that in the absence of SP-A, sex has very little impact, if any, on the BAL cellular composition and markers of tissue damage and PMN recruitment under the studied experimental conditions and may not significantly contribute to the previously observed sex differences in survival [4,5].

We also measured the total phospholipid levels because of their role in the modulation of SP-A host defense [53,54] and alveolar macrophage activities [13,55]. Infection alone had no impact on phospholipid levels, but there was a progressive increase in phospholipid levels as early as 4 h following O_3_ exposure. Compared to WT mice [30], lipid levels were much higher in SP-A KO mice following O_3_ exposure in both males and females. SP-A was shown to inhibit surfactant phospholipid secretion after LPS-induced lung injury [56]; therefore, it was not surprising to observe higher phospholipids in SP-A KO mice compared to the WT mice. At the early stage (first 24 h post-infection or O_3_ exposure), the tubular myelin, an extracellular form of surfactant present in alveolar surface fluid in WT mice, may release surfactant lipids and proteins, but the SP-A KO mice lack this mechanism [57]. What is of interest, however, is that at the 48 h time point after O_3_ exposure, a significant difference between males and females was observed in both WT [30] and KO mice (present study), thus indicating that the observed sex difference is independent of SP-A. Furthermore, higher lipid levels have been shown to inhibit the respiratory burst [58], and this may impair the ability of macrophages to clear infection and/or oxidative stress, thus increasing disease severity and lower survival of SP-A KO mice compared to WT mice following ozone exposure and infection [4,5]. Additionally, ozone-induced epithelial damage in KO mice, as assessed by increased LDH, may have contributed to the observed phospholipid increase in BAL.

In addition to the immune response [59], sex hormones have been shown to influence lung function, the course of disease, and the response to environmental agents (e.g., O_3_) in animal models and in humans [21]. E_2_ is the major form of estrogen and DHT is the active form of testosterone in adult females and males, respectively [59]. In the current study, Gx females and Gx males received DHT and E_2_, respectively. This was done in order to better elucidate the effects of circulating gonadal hormones on survival in response to infection with or without prior O_3_ exposure that would be independent of chromosomal gender, sex-dependent anatomic and physiologic airway differences, and/or developmental programming.

Previous data generated in our laboratory have shown that the daily survival of O_3_-exposed WT and SP-A KO male and female animals are significantly reduced compared to the corresponding FA-exposed groups, as well as that females are at higher risk [4,5]. In the present study, we observed that gonadectomy of SP-A KO FA-exposed male and female mice eliminated previously observed sex differences in survival (Figure 5B,C) [4,22]. Furthermore, no sex differences were observed in the phagocytic index between Gx male and Gx female KO FA-exposed mice, a finding similar to that in intact SP-A KO mice (Figure 5A). However, the treatment of Gx male and female SP-A KO mice with E_2_ and DHT, respectively, did not restore the difference in the survival rates of FA- and O_3_-exposed mice to those of the intact SP-A KO male and female mice, when males and females were analyzed separately (data not shown). In contrast, the gonadectomy of WT mice eliminated (in females) and/or reduced (in males) the impact of O_3_ exposure on the survival of Gx WT mice compared to FA-exposed mice [24], and treatment with hormones restored that difference to that of the intact WT mice. This sex-dependent survival restoration occurred with a dataset that had a similar sample size (*n* = 25/group) as that of the present study [24]. Previously, females have shown a survival advantage in response to *K. pneumoniae* infection alone (FA-exposed), but prior O_3_ exposure reversed this pattern (males > females) in WT and non-Gx SP-A KO mice [4,22,24]. In both of these studies [4,5], as well as in a study of infected SP-A KO (without ozone exposure) [22], a much larger sample size was used. Whether the finding of no sex differences in the current study was due to the smaller sample size (~25 animals/group) compared to the previously published studies (~50–80 animals/group) [4,22] remains to be determined. However, sex differences with smaller sample size (*n* = ~25) have been observed in Gx WT [24] and humanized transgenic mice, where each expressed a different SP-A variant [22]. Together, these data indicate that if there is any sex difference in Gx SP-A KO mice, this difference must be very small.

To further understand the interactions of gonadal hormones, SP-A, and sex on survival after infection in the presence or absence of O_3_ exposure, we compared the mean survival data of the current study with the previously published data of intact (non-Gx) SP-A KO and wild type mice [4,24]. As shown in Figure 7, the gonadectomy of SP-A KO FA- and O_3_-exposed mice improved survival to levels greater than that of intact SP-A KO mice in both sexes. However, the treatment of GxF and GxM mice with DHT and E_2_, respectively, did not change the survival rate to that observed in the intact SP-A KO mice [4]. These findings, as noted above, were in contrast to Gx WT study where the replacement of hormones changed the mean survival rate to levels similar to those in the intact WT mice [24]. Conversely, FA- and O_3_-exposed SP-A KO GxF and DHT mice in the current study showed a similar survival pattern to that of the GxM and CoP mice (Figure 7). Of note, optimal sex hormone levels determine immune response (anti- or pro-inflammatory) to various insults such as viruses and bacteria [60]. Moreover, in humans, an association of lower serum DHT levels with a favorable long-term survival after pneumonia has been observed in males [61]. As mentioned above, gonadal hormones have a limited impact on sex-dependent survival in the absence of SP-A; therefore, it is possible that the DHT dose used to treat the GxF mice in the current study may not have been enough to reduce survival to the level of the intact SP-A KO males, although the same dose changed the survival of the Gx WT females to that of the intact WT male [24]. Conversely, because very low levels of DHT were detected in the Gx WT male mice [24], it is possible that this low level of DHT in the absence of SP-A exhibited the same impact on survival as the higher level of DHT achieved via the hormone treatment.

Though no difference in the survival rate of the FA-exposed GxM and E_2_ mice and the GxF and CoP mice was observed, the reduced survival in response to O_3_ exposure of the GxM and E_2_ mice vs that of the GxF and CoP SP-A KO mice indicated the detrimental effect of estrogen on survival if infection is preceded by ozone exposure. This observation was in line with previous animal and human studies showing severe respiratory diseases in females exposed to air pollution and oxidative stress compared to males [4,5,29,30,31,62,63].

An unexpected and surprising observation was that not only the overall survival is improved in the Gx KO mice, either in response to FA or O_3_, compared to the intact KO, but this improved survival rate was similar or approximating that observed in intact WT animals. This observation was rather puzzling. It has been shown that in the absence of SP-A, the survival of infected mice decreases in both FA and O_3_ compared to WT mice. Moreover, under similar conditions in the absence of gonadal hormones, as in the case of the Gx WT mice, the survival rate increased in the Gx WT males (FA, O_3_) and females (O_3_) and was reduced in FA females. In the present study, where both SP-A and gonadal hormones were absent, the survival was remarkably high, closer to that of WT mice but without detectable sex differences, in the overall survival (Figure 7). Collectively, the published and present data indicate that gonadal hormones play an important role in determining sex-specific survival in the presence or absence of SP-A. However, when both gonadal hormones and SP-A are absent (present study), sex-specific survival is largely eliminated, thus indicating that SP-A may directly or indirectly contribute to sex-specific health outcomes. In the presence of ozone, however, a decreased survival was observed in the GxM and E_2_ mice vs the GxF and CoP mice, thus indicating that ozone exposure has a negative impact on female survival, independent of SP-A. This finding is consistent with observations made for WT [5] and SP-A KO mice [4]. In humans, a decreased level of SP-A has been found in the BAL of patients admitted with Gram-positive bacterial pneumonia [64]. The absence or functional impairment of SP-A may be contributing to an increased risk of hospitalization due to pneumonia when ambient ozone levels are high [65,66], and SP-A proteins or perhaps peptides of SPs can be used as modulatory contributors to innate immune function against bacterial pneumonia in the future.

## 5. Conclusions

In summary, the collective data indicate that in the absence of SP-A: (a) no major significant sex-specific changes occurred in several BAL readouts at 4, 24, and 48 h post-infection, thus indicating a role of SP-A in sex-specific differences; (b) the prior ozone exposure of infected mice resulted in significant increases or decreases of the studied readouts in BAL in most of the post-infection time points, thus indicating a role of ozone in these parameters. The changed studied parameters included cellular distribution (i.e., % of PMNs and monocytes), total protein concentration and total oxidized protein content, markers of cellular damage (LDH, MIP-2, and total phospholipids); (c) in the absence of SP-A and gonadal hormones, sex-specificity in survival is eliminated; and (d) ozone has a significant negative impact on the survival of Gx mice with or without hormone replacement when compared to the Gx mice exposed to FA, though an exception was seen for the GxF and CoP mice. Moreover, ozone exposure was found to have a negative impact on the survival of Gx mice with E_2_ replacement compared to controls (i.e., Gx mice with CoP), thus indicating an interaction of female hormones and ozone. We postulate that different mechanisms are partly operative in the presence and absence of SP-A, via which gonadal hormones bring about sex-specific survival.

## References

[1] World Health Organization (2012). Pneumococcal vaccines: WHO position paper—2012. Wkly. Epidemiol. Rec. Relev. Épidémiologique Hebd..

[2] Troeger C., Forouzanfar M., Rao P.C., Khalil I., Brown A., Swartz S., Fullman N., Mosser J., Thompson R.L., Reiner R.C. (2017). Estimates of the global, regional, and national morbidity, mortality, and aetiologies of lower respiratory tract infections in 195 countries: A systematic analysis for the Global Burden of Disease Study 2015. Lancet Infect. Dis..

[3] Paganin F., Lilienthal F., Bourdin A., Lugagne N., Tixier F., Genin R., Yvin J. (2004). Severe community-acquired pneumonia: Assessment of microbial aetiology as mortality factor. Eur. Respir. J..

[4] Mikerov A.N., Haque R., Gan X., Guo X., Phelps D.S., Floros J. (2008). Ablation of SP-A has a negative impact on the susceptibility of mice to Klebsiella pneumoniae infection after ozone exposure: Sex differences. Respir. Res..

[5] Mikerov A.N., Gan X., Umstead T.M., Miller L., Chinchilli V.M., Phelps D.S., Floros J. (2008). Sex differences in the impact of ozone on survival and alveolar macrophage function of mice after Klebsiella pneumoniae infection. Respir. Res..

[6] Baleeiro C.E., Wilcoxen S.E., Morris S.B., Standiford T.J., Paine R. (2003). Sublethal hyperoxia impairs pulmonary innate immunity. J. Immunol..

[7] Laichalk L.L., Kunkel S.L., Strieter R.M., Danforth J.M., Bailie M.B., Standiford T.J. (1996). Tumor necrosis factor mediates lung antibacterial host defense in murine Klebsiella pneumonia. Infect. Immun..

[8] Markart P., Korfhagen T.R., Weaver T.E., Akinbi H.T. (2004). Mouse lysozyme M is important in pulmonary host defense against Klebsiella pneumoniae infection. Am. J. Respir. Crit. Care Med..

[9] Cogen A.L., Moore T.A. (2009). β2-Microglobulin-Dependent Bacterial Clearance and Survival during Murine Klebsiella pneumoniae Bacteremia. Infect. Immun..

[10] Ketko A.K., Donn S.M. (2014). Surfactant-associated proteins: Structure, function and clinical implications. Curr. Pediatr. Rev..

[11] Crouch E., Hartshorn K., Ofek I. (2000). Collectins and pulmonary innate immunity. Immunol. Rev..

[12] Nathan N., Taytard J., Duquesnoy P., Thouvenin G., Corvol H., Amselem S., Clement A. (2016). Surfactant protein A: A key player in lung homeostasis. Int. J. Biochem. Cell Biol..

[13] Phelps D.S. (2001). Surfactant regulation of host defense function in the lung: A question of balance. Pediatric Pathol. Mol. Med..

[14] Brinker K.G., Garner H., Wright J.R. (2003). Surfactant protein A modulates the differentiation of murine bone marrow-derived dendritic cells. Am. J. Physiol. Lung Cell. Mol. Physiol..

[15] Kremlev S.G., Phelps D.S. (1997). Effect of SP-A and surfactant lipids on expression of cell surface markers in the THP-1 monocytic cell line. Am. J. Physiol. Lung Cell. Mol. Physiol..

[16] Bridges J.P., Davis H.W., Damodarasamy M., Kuroki Y., Howles G., Hui D.Y., McCormack F.X. (2000). Pulmonary surfactant proteins A and D are potent endogenous inhibitors of lipid peroxidation and oxidative cellular injury. J. Biol. Chem..

[17] Crowther J.E., Kutala V.K., Kuppusamy P., Ferguson J.S., Beharka A.A., Zweier J.L., McCormack F.X., Schlesinger L.S. (2004). Pulmonary surfactant protein a inhibits macrophage reactive oxygen intermediate production in response to stimuli by reducing NADPH oxidase activity. J. Immunol..

[18] Hickman-Davis J.M., Gibbs-Erwin J., Lindsey J.R., Matalon S. (2004). Role of surfactant protein-A in nitric oxide production and mycoplasma killing in congenic C57BL/6 mice. Am. J. Respir. Cell Mol. Biol..

[19] LeVine A.M., Kurak K.E., Bruno M.D., Stark J.M., Whitsett J.A., Korfhagen T.R. (1998). Surfactant protein-A-deficient mice are susceptible to Pseudomonas aeruginosa infection. Am. J. Respir. Cell Mol. Biol..

[20] LeVine A.M., Whitsett J.A., Gwozdz J.A., Richardson T.R., Fisher J.H., Burhans M.S., Korfhagen T.R. (2000). Distinct effects of surfactant protein A or D deficiency during bacterial infection on the lung. J. Immunol..

[21] Card J.W., Zeldin D.C. (2009). Hormonal influences on lung function and response to environmental agents: Lessons from animal models of respiratory disease. Proc. Am. Thorac. Soc..

[22] Thorenoor N., Umstead T.M., Zhang X., Phelps D.S., Floros J. (2018). Survival of Surfactant Protein-A1 and SP-A2 Transgenic Mice After Klebsiella pneumoniae Infection, Exhibits Sex-, Gene-, and Variant Specific Differences; Treatment with Surfactant Protein Improves Survival. Front. Immunol..

[23] Thorenoor N., Zhang X., Umstead T.M., Scott Halstead E., Phelps D.S., Floros J. (2018). Differential effects of innate immune variants of surfactant protein-A1 (SFTPA1) and SP-A2 (SFTPA2) in airway function after Klebsiella pneumoniae infection and sex differences. Respir. Res..

[24] Durrani F., Phelps D.S., Weisz J., Silveyra P., Hu S., Mikerov A.N., Floros J. (2012). Gonadal hormones and oxidative stress interaction differentially affects survival of male and female mice after lung Klebsiella pneumoniae infection. Exp. Lung Res..

[25] Mikerov A.N., Hu S., Durrani F., Gan X., Wang G., Umstead T.M., Phelps D.S., Floros J. (2012). Impact of sex and ozone exposure on the course of pneumonia in wild type and SP-A (-/-) mice. Microb. Pathog..

[26] Kaplan V., Angus D.C., Griffin M.F., Clermont G., Scott Watson R., Linde-Zwirble W.T. (2002). Hospitalized community-acquired pneumonia in the elderly: Age-and sex-related patterns of care and outcome in the United States. Am. J. Respir. Crit. Care Med..

[27] Gannon C.J., Pasquale M., Tracy J.K., McCarter R.J., Napolitano L.M. (2004). Male gender is associated with increased risk for postinjury pneumonia. Shock.

[28] Card J.W., Carey M.A., Bradbury J.A., DeGraff L.M., Morgan D.L., Moorman M.P., Flake G.P., Zeldin D.C. (2006). Gender differences in murine airway responsiveness and lipopolysaccharide-induced inflammation. J. Immunol..

[29] Mikerov A.N., Cooper T.K., Wang G., Hu S., Umstead T.M., Phelps D.S., Floros J. (2011). Histopathologic evaluation of lung and extrapulmonary tissues show sex differences in Klebsiella pneumoniae-infected mice under different exposure conditions. Int. J. Physiol. Pathophysiol. Pharmacol..

[30] Mikerov A.N., Phelps D.S., Gan X., Umstead T.M., Haque R., Wang G., Floros J. (2014). Effect of ozone exposure and infection on bronchoalveolar lavage: Sex differences in response patterns. Toxicol. Lett..

[31] Dye J.A., Gibbs-Flournoy E.A., Richards J.H., Norwood J., Kraft K., Hatch G.E. (2017). Neonatal rat age, sex and strain modify acute antioxidant response to ozone. Inhal. Toxicol..

[32] Ikegami M., Elhalwagi B.M., Palaniyar N., Dienger K., Korfhagen T., Whitsett J.A., McCormack F.X. (2001). The Collagen-like Region of Surfactant Protein A (SP-A) Is Required for Correction of Surfactant Structural and Functional Defects in the SP-A Null Mouse. J. Biol. Chem..

[33] Wang G., Guo X., Diangelo S., Thomas N.J., Floros J. (2010). Humanized SFTPA1 and SFTPA2 transgenic mice reveal functional divergence of SP-A1 and SP-A2: Formation of tubular myelin in vivo requires both gene products. J. Biol. Chem..

[34] Schmiedl A., Vieten G., Mühlfeld C., Bernhard W. (2007). Distribution of intracellular and secreted surfactant during postnatal rat lung development. Pediatric Pulmonol..

[35] Oosting R., Van Greevenbroek M., Verhoef J., Van Golde L., Haagsman H. (1991). Structural and functional changes of surfactant protein A induced by ozone. Am. J. Physiol. Lung Cell. Mol. Physiol..

[36] Wang G., Bates-Kenney S.R., Tao J.Q., Phelps D.S., Floros J. (2004). Differences in biochemical properties and in biological function between human SP-A1 and SP-A2 variants, and the impact of ozone-induced oxidation. Biochemistry.

[37] Wang G., Umstead T.M., Phelps D.S., Al-Mondhiry H., Floros J. (2002). The effect of ozone exposure on the ability of human surfactant protein a variants to stimulate cytokine production. Environ. Health Perspect..

[38] Huang W., Wang G., Phelps D.S., Al-Mondhiry H., Floros J. (2004). Human SP-A genetic variants and bleomycin-induced cytokine production by THP-1 cells: Effect of ozone-induced SP-A oxidation. Am. J. Physiol. Lung Cell. Mol. Physiol..

[39] Oosting R.S., Iwaarden J.F.V., Bree L.V., Verhoef J., Golde L.M.V., Haagsman H.P. (1992). Exposure of surfactant protein A to ozone in vitro and in vivo impairs its interactions with alveolar cells. Am. J. Physiol. Lung Cell. Mol. Physiol..

[40] Mikerov A.N., Umstead T.M., Gan X., Huang W., Guo X., Wang G., Phelps D.S., Floros J. (2008). Impact of ozone exposure on the phagocytic activity of human surfactant protein A (SP-A) and SP-A variants. Am. J. Physiol. Lung Cell. Mol. Physiol..

[41] Wang G., Myers C., Mikerov A., Floros J. (2007). Effect of cysteine 85 on biochemical properties and biological function of human surfactant protein A variants. Biochemistry.

[42] Delgado-Roche L., Riera-Romo M., Mesta F., Hernández-Matos Y., Barrios J.M., Martínez-Sánchez G., Al-Dalaien S.M. (2017). Medical ozone promotes Nrf2 phosphorylation reducing oxidative stress and pro-inflammatory cytokines in multiple sclerosis patients. Eur. J. Pharm..

[43] Wang G., Umstead T.M., Hu S., Mikerov A.N., Phelps D.S., Floros J. (2019). Differential Effects of Human SP-A1 and SP-A2 on the BAL Proteome and Signaling Pathways in Response to Klebsiella pneumoniae and Ozone Exposure. Front. Immunol..

[44] Haque R., Umstead T.M., Ponnuru P., Guo X., Hawgood S., Phelps D.S., Floros J. (2007). Role of surfactant protein-A (SP-A) in lung injury in response to acute ozone exposure of SP-A deficient mice. Toxicol. Appl. Pharm..

[45] Mikerov A.N., Umstead T.M., Huang W., Liu W., Phelps D.S., Floros J. (2005). SP-A1 and SP-A2 variants differentially enhance association of Pseudomonas aeruginosa with rat alveolar macrophages. Am. J. Physiol. Lung Cell. Mol. Physiol..

[46] Mikerov A.N., Wang G., Umstead T.M., Zacharatos M., Thomas N.J., Phelps D.S., Floros J. (2007). Surfactant protein A2 (SP-A2) variants expressed in CHO cells stimulate phagocytosis of Pseudomonas aeruginosa more than do SP-A1 variants. Infect. Immun..

[47] LeVine A.M., Hartshorn K., Elliott J., Whitsett J., Korfhagen T. (2002). Absence of SP-A modulates innate and adaptive defense responses to pulmonary influenza infection. Am. J. Physiol. Lung Cell. Mol. Physiol..

[48] Li G., Siddiqui J., Hendry M., Akiyama J., Edmondson J., Brown C., Allen L., Levitt S., Poulain F., Hawgood S. (2002). Surfactant Protein-A–Deficient Mice Display an Exaggerated Early Inflammatory Response to a β -Resistant Strain of Influenza A Virus. Am. J. Respir. Cell Mol. Biol..

[49] Balamayooran G., Batra S., Fessler M.B., Happel K.I., Jeyaseelan S. (2010). Mechanisms of neutrophil accumulation in the lungs against bacteria. Am. J. Respir. Cell Mol. Biol..

[50] Floros J., Phelps D., Nakos G., Lekka M. (2002). Surfactant-update of intensive care medicine. Update of Intensive Care Medicine.

[51] LeVine A.M., Kurak K.E., Wright J.R., Watford W.T., Bruno M.D., Ross G.F., Whitsett J.A., Korfhagen T.R. (1999). Surfactant Protein-A Binds Group B Streptococcus Enhancing Phagocytosis and Clearance from Lungs of Surfactant Protein-A–Deficient Mice. Am. J. Respir. Cell Mol. Biol..

[52] LeVine A.M., Gwozdz J., Stark J., Bruno M., Whitsett J., Korfhagen T. (1999). Surfactant protein-A enhances respiratory syncytial virus clearance in vivo. J. Clin. Investig..

[53] Koptides M., Umstead T.M., Floros J., Phelps D.S. (1997). Surfactant protein A activates NF-kappa B in the THP-1 monocytic cell line. Am. J. Physiol. Lung Cell. Mol. Physiol..

[54] Song M., Phelps D.S. (2000). Comparison of SP-A and LPS effects on the THP-1 monocytic cell line. Am. J. Physiol. Lung Cell. Mol. Physiol..

[55] Floros J., Wang G., Mikerov A.N. (2009). Genetic complexity of the human innate host defense molecules, surfactant protein A1 (SP-A1) and SP-A2--impact on function. Crit. Rev. Eukaryot. Gene Exp..

[56] Quintero O.A., Korfhagen T.R., Wright J.R. (2002). Surfactant protein A regulates surfactant phospholipid clearance after LPS-induced injury in vivo. Am. J. Physiol. Lung Cell. Mol. Physiol..

[57] Pérez-Gil J. (2008). Structure of pulmonary surfactant membranes and films: The role of proteins and lipid–protein interactions. Biochim. Et Biophys. Acta (BBA) Biomembr..

[58] Tonks A., Parton J., Tonks A.J., Morris R.H.K., Finall A., Jones K.P., Jackson S.K. (2005). Surfactant phospholipid DPPC downregulates monocyte respiratory burst via modulation of PKC. Am. J. Physiol. Lung Cell. Mol. Physiol..

[59] Frump A.L., Lahm T. (2016). Sex hormone signaling in the lung in health and disease: Airways, parenchyma, and pulmonary vasculature. Gender, Sex Hormones and Respiratory Disease.

[60] Taneja V. (2018). Sex Hormones Determine Immune Response. Front. Immunol..

[61] Zurfluh S., Nickler M., Ottiger M., Steuer C., Kutz A., Christ-Crain M., Zimmerli W., Thomann R., Hoess C., Henzen C. (2018). Dihydrotestosterone is a predictor for mortality in males with community-acquired pneumonia: Results of a 6-year follow-up study. Respir. Res..

[62] Lauritzen S.K., Adams W.C. (1985). Ozone inhalation effects consequent to continuous exercise in females: Comparison to males. J. Appl. Physiol..

[63] Di Q., Wang Y., Zanobetti A., Wang Y., Koutrakis P., Choirat C., Dominici F., Schwartz J.D. (2017). Air Pollution and Mortality in the Medicare Population. N. Engl. J. Med..

[64] Baughman R.P., Sternberg R.I., Hull W., Buchsbaum J.A., Whitsett J. (1993). Decreased surfactant protein A in patients with bacterial pneumonia. Am. Rev. Respir. Dis..

[65] Ji W., Park Y.R., Kim H.R., Kim H.-C., Choi C.-M. (2017). Prolonged effect of air pollution on pneumonia: A nationwide cohort study. Eur. Respir. J..

[66] Croft D.P., Zhang W., Lin S., Thurston S.W., Hopke P.K., Masiol M., Squizzato S., Van Wijngaarden E., Utell M.J., Rich D.Q. (2019). The Association between Respiratory Infection and Air Pollution in the Setting of Air Quality Policy and Economic Change. Ann. Am. Thorac. Soc..

